# Medical Gleanings in Naples

**Published:** 1851-01

**Authors:** 


### ARTICLE IX.

*Medical Gleanings in Naples. From the Editorial Corres dence of the Boston Medical Journal.*

The Neapolitians entertain an opinion that bloodletting is indicated in many diseases in which, among us, it would be thought fatal. Bleeding is a distinct profession and in narrow lanes, it is quite common to find painted signs, representing a nude man, tapped at several points—a stream of blood-flowing from the arm, the neck and foot, all at the same moment. In the spring, everybody is supposed to require bleeding, just as, in some parts of New England, whole neighborhoods at that season take physic. Horses, too, are here bled unmercifully. A few days since, a poor, overworked creature was standing in the middle of the street, his blood flowing out with frightful rapidity. He required food, instead of cruel depletion. Consumption is considered infectious; consequently, on the death of a person fiom pulmonary disease, his clothes are burned and the apartment at once thoroughly purified. An instance was related by a high public functionary, the other day, of a family being warued to vac-te their hired premises, forthwith, because a member of the family gave indications of approaching pulmonary consumption. Nowhere are the dead more magnificently exhibited at a funeral, or more quickly disposed of when the ceremonies are finished. One coffin answers for thousands, to all appearance. It is of rough, white boards—lodged temporarily, while in the church, in a rich sarcophagus, covered by a richly wrought pall, made heavy by gold lace and fringes. When the candles are extinguished the friends retire, and the coffin being taken out, is carried on the heads of rough-looking fellows to a closet. Afterwards, if conveyed to the Santo Campo, the corpse is taken out of the coffin and laid on a shelf in a tomb, and the empty box brought back for another. Some of the funeral processions in Naples, Rome and Florence are very extraordinary performances—the persons following are all masked, having eyeholes to see through, while by-standers are prevented from recognizing any of them. At Florence the burials are by night.

Naples, Oct. 22, 1850.

